# Effects of outdoor sports on college students’ learning burnout: a multiple mediation model of social support and self-regulation

**DOI:** 10.3389/fpsyg.2025.1629986

**Published:** 2025-10-24

**Authors:** Peng Xu

**Affiliations:** School of Leisure Sports, Chengdu Sport University, Chengdu, Sichuan, China

**Keywords:** outdoor sports, learning burnout, social support, self-regulation ability, university students, mediation model

## Abstract

**Objective:**

This study investigates the alleviating effect of outdoor sports on learning burnout among university students, focusing on the mediating roles of social support and self-regulation ability.

**Methods:**

A cross-sectional survey was conducted with 942 university students in China. Structural Equation Modeling (SEM) was employed to explore the direct and indirect effects of outdoor sports on learning burnout.

**Results:**

The results revealed that outdoor sports negatively predicted learning burnout both directly and indirectly. Social support and self-regulation ability were found to partially mediate this relationship. Additionally, a significant chain mediation effect was identified, where outdoor sports enhanced social support, which in turn fostered self-regulation, ultimately reducing learning burnout.

**Conclusion:**

The findings highlight the importance of integrating outdoor sports into mental health and academic support strategies. This study contributes to a deeper understanding of how external and internal psychological resources interact to reduce academic stress and promote well-being.

## 1 Introduction

Learning burnout, as a common psychological phenomenon in higher education, has attracted increasing attention from both the fields of education and psychology. It is mainly manifested as emotional exhaustion, reduced academic interest, and a decline in efficacy, and is often associated with sustained academic stress and difficulties in contextual adaptation (Luo et al., 2024; Wu et al., 2010). Research has shown that learning burnout not only undermines students’ academic performance but may also trigger psychological problems such as anxiety and depression, ultimately affecting their overall quality of life and developmental potential (Ying et al., 2023; Zhang et al., 2024, 2025). In the context of intensified competition and increasing academic workload, identifying effective coping pathways has become an urgent issue within university education systems.

Compared with primary and secondary school students, college students exhibit unique developmental and learning characteristics. They are in a critical transitional stage from adolescence to adulthood, during which they must cope simultaneously with academic pressure, career preparation, interpersonal relationships, and identity development (Zhao et al., 2023). The increase in autonomy at this stage makes them more independent in time management and emotional regulation, yet also more vulnerable to psychological difficulties due to limited experience and insufficient external support (Griffin et al., 2025). Therefore, college students are not only a high-risk group for learning burnout but also a key population for relevant intervention research.

Outdoor sports, which integrates physical activity, exposure to nature, and situational experiences, has gradually become a focus in psychological interventions and quality education in universities. Existing studies suggest that natural environments exert positive effects on emotional restoration (Marselle et al., 2013), and that moderate outdoor sports helps to relieve stress and improve psychological well-being (Calogiuri et al., 2023; Tang et al., 2024). In campus practice, diverse forms of outdoor activities have been incorporated into mental health support programs, expanding the scope of interventions beyond traditional classrooms. Nevertheless, the mechanisms through which outdoor sports influences students’ learning states remain underexplored, and systematic investigation of its role in alleviating learning burnout is still lacking.

In student development research, social support has long been recognized as an important resource for promoting psychological adaptation. Positive support from family, peers, and teachers can enhance students’ sense of security, belonging, and coping capacity when facing difficulties (Chen et al., 2025; Rahat and İlhan, 2016; Xu et al., 2024). With the improvement of psychological service systems in universities, the forms and functions of social support have become increasingly diverse, and its expression in informal educational contexts has gradually emerged as a new research domain (Heerde and Hemphill, 2018). Particularly in collective activities and interactive settings, the perception and construction of social support have shown more complex characteristics (Sheridan et al., 2014), warranting further analysis in practical contexts.

Self-regulation, defined as the psychological mechanism by which individuals manage their cognition, emotions, and behaviors to achieve goals (Hall and Fong, 2007), serves as a crucial indicator of university students’ ability to grow independently and adapt to external pressures. In the learning process, self-regulation encompasses dimensions such as planning ability, motivational control, and emotional regulation, and has been widely used to explain persistence in learning and resilience to stress (Jixia, 2008; Wu, 2008). In recent years, research has begun to focus on how participatory and experiential activities can foster the development of students’ self-regulation (Ahn et al., 2016; Song and Lee, 2021), suggesting that incorporating daily activities into capacity-building systems is practically feasible. Exploring the role of self-regulation in the context of academic stress not only enriches theoretical perspectives but also provides more targeted strategies for psychological support for college students.

Taken together, the severity and multidimensional impact of learning burnout call for the integration of cross-disciplinary resources to identify more effective coping approaches. Current research on the role of outdoor sports in alleviating learning burnout remains limited, especially with regard to its underlying mechanisms. Building on existing findings, this study employs empirical methods to further explore the psychological resources involved in outdoor sports practices, thereby providing theoretical references and practical implications for mental health support strategies and campus physical activity design in higher education.

## 2 Literature review and research hypotheses

### 2.1 The relationship between outdoor sports and college students’ learning burnout

Learning burnout refers to a psychological state in which individuals gradually lose interest in learning, experience emotional exhaustion, and perceive a decline in learning efficiency due to prolonged academic pressure (Chen et al., 2022; Lin and Fengyan, 2023; Hongyi and Fengyan, 2023; Jianwen et al., 2022). This concept was first introduced by Schaufeli et al. (2002) and has been widely applied in the field of educational psychology to describe the negative emotional responses students experience in high-pressure academic environments. Research generally suggests that learning burnout is not only influenced by external factors, such as academic burden and time management difficulties, but also closely related to internal factors like emotional regulation ability, cognitive resource depletion, and self-efficacy (Hong and Hanafi, 2024; Ma, 2024; Sun et al., 2024; Zhu, 2025). As research progresses, scholars have started to focus on intervention strategies for this issue, such as optimizing the learning environment, enhancing psychological support systems, and improving students’ self-regulation abilities (Yang et al., 2022).

Emotion regulation theory posits that physical exercise provides a channel for emotional expression and regulation, allowing individuals to cope with external pressures in a more positive way (Gross, 1998; Wu and Zhou, 2024). Emotion regulation refers to “the process by which individuals influence their emotions, when they have them, and how they experience and express these emotions” (Gross, 1999). In the academic context, students often face academic pressure and high-intensity academic tasks. A lack of effective emotion regulation may lead to emotional exhaustion and the development of learning burnout (Iuga and David, 2024). Existing studies have shown that physical exercise, especially for adolescents, serves as an effective emotion regulation tool, increasing self-efficacy and alleviating negative emotional fluctuations, helping students effectively manage academic pressure, and improving emotional recovery, thereby reducing learning burnout (Fu et al., 2023). However, most studies have primarily focused on adolescent populations (Tong et al., 2023), with fewer studies involving college students. Therefore, this study aims to explore how outdoor sports, a specific form of physical exercise, influences college students’ learning burnout through emotion regulation mechanisms, and to investigate the specific pathways by which outdoor sports affect college students’ academic burnout.

Outdoor sports, as an activity combining physical exercise and psychological regulation, have gradually attracted attention from mental health researchers. Existing studies have shown that outdoor sports not only help improve physical fitness but also effectively promote emotion regulation and alleviate individuals’ negative emotional experiences (Guarda-Saavedra et al., 2022; Marcos-Pardo et al., 2024). Participants in such activities typically experience positive emotional changes, such as reduced anxiety, emotional recovery, and an increased sense of calm, which improve their psychological state and enhance their subjective well-being (Bramwell et al., 2023; Wicks et al., 2022). For college students, outdoor sports provide a temporary escape from academic pressure, allowing them to experience emotional release and psychological recovery through contact with nature and physical activity (Baker, 2024). Additionally, research indicates that the social interaction and self-challenge elements in outdoor sports can enhance an individual’s self-efficacy (Mutz and Müller, 2016), which may play a positive role in addressing self-doubt and motivation loss during academic tasks. Based on existing literature, outdoor sports have a positive impact on college students’ emotion regulation and mental health, and may be a beneficial way to alleviate learning burnout. During exercise, students not only achieve physical relaxation but also restore psychological energy at the cognitive and emotional levels, thereby alleviating learning burnout caused by academic stress.

*H1*: Outdoor sports are hypothesized to be negatively associated with learning burnout.

### 2.2 Exploration of mediating variables between outdoor sports and college students’ learning burnout

#### 2.2.1 Social support

Social support refers to the emotional, informational, and practical assistance that individuals receive from their social networks when facing life challenges (Buunk and Hoorens, 1992; Cobb, 1976). Social support is widely recognized as one of the key factors in emotional regulation and psychological adaptation (Lopez et al., 2024). Research has shown that social support can alleviate stress and negative emotions through various pathways, thereby enhancing psychological well-being (Guo et al., 2020; Yang et al., 2023). In high-pressure academic environments, college students often face academic burdens and time management pressures, which can lead to emotional exhaustion and a decline in academic motivation. However, social support helps students better cope with these pressures by providing emotional comfort, practical help, and encouragement, thereby stabilizing emotions and effectively reducing the negative impact of academic stress (Ullah et al., 2023). Social support not only directly aids students’ psychological recovery but may also enhance their self-efficacy, helping them better cope with academic challenges (Ozsaker et al., 2015; Ren et al., 2020).

Social support theory suggests that support from others (such as peers, teachers, and family members) helps individuals cope with life stress, thus promoting psychological health (Acoba, 2024). This role is particularly prominent in collective activities, where social interaction and support networks become even more important. For college students, the social interaction and self-challenge elements in outdoor sports not only help with emotional recovery but also enhance students’ self-efficacy (Tyne et al., 2024). Moreover, the mediating role of social support has been supported in existing studies, which discuss how social support mediates the relationship between sports participation and learning burnout in adolescents (Gao et al., 2025). Therefore, social support in this process may provide emotional support to students, helping them alleviate academic stress and psychological burdens, thereby promoting learning adaptation and psychological recovery.

In the process of sports participation, social support also plays an important role. Many studies have found that individuals who engage in sports, especially in group or team activities, typically receive emotional and practical support from peers, team members, and social circles (Paldi et al., 2021; Zhao et al., 2022). This support not only helps improve emotional states but also boosts motivation to participate. In collective sports activities, social interaction and emotional connection become important driving forces for individual participation (Hwang et al., 2017). Through these interactions, athletes receive the necessary support to more effectively cope with the physical exertion and psychological challenges of sports, thereby improving the sustainability and involvement of the activity. For college students, sports not only provide an outlet for academic stress but also offer a temporary escape from intense academic tasks, allowing for physical and mental recovery (Monserrat-Hernández et al., 2023; Zhu et al., 2024). In this process, social support may play a significant role in the relationship between outdoor sports and learning burnout. By alleviating students’ emotional stress, enhancing their resilience and sense of social belonging, social support can help students reduce the psychological burdens of academic tasks, thereby promoting their mental health and academic adaptation.

*H2*: Social support statistically mediates the association between outdoor sports and learning burnout.

#### 2.2.2 Self-regulation ability

Self-regulation ability refers to an individual’s ability to actively control their emotions, thoughts, and behaviors in goal-directed activities (Ren et al., 2023). This ability typically includes aspects such as emotional control, attention maintenance, motivation regulation, and behavioral adjustment (Davis et al., 2020; Heatherton, 2011). Research shows that self-regulation ability helps individuals maintain psychological stability and behavioral consistency when facing complex tasks and stressful situations, thus enhancing adaptability (Park et al., 2012). In the context of higher education, university students often face continuous academic pressure and cognitive load. Without effective self-regulation ability, they are more likely to experience learning burnout, distractibility, and decreased motivation. In contrast, individuals with strong self-regulation ability are better able to cope with academic challenges through goal management, time scheduling, and emotional adjustment, reducing the negative impact of prolonged academic tasks on their mental state (Ibrahim et al., 2024). Additionally, previous studies have shown that self-regulation ability is closely related to an individual’s learning motivation, self-efficacy, and stress resilience (Vent et al., 2021; Yu et al., 2022), making it a key factor in academic success and mental health.

Self-regulation theory suggests that self-regulation ability helps individuals control their emotions, maintain focus, and effectively complete tasks when facing stress (van den Bekerom et al., 2025). Among university students, academic pressure and learning burnout are common issues, and students with strong self-regulation abilities can reduce the negative impact of academic tasks on their mental health through effective emotional regulation (de la Fuente et al., 2020). Moreover, outdoor sports are considered an effective way to develop self-regulation abilities. The emotional regulation and psychological recovery functions of physical exercise help university students maintain motivation and emotional stability under academic pressure, thereby better coping with challenges in their studies (Zhu et al., 2025).

In the context of physical activity participation, self-regulation ability is also considered an important variable influencing exercise persistence and psychological adaptation. Research shows that outdoor sports help alleviate stress, release frustration, adjust emotions, and maintain good psychological states (Chong et al., 2013). When individuals participate in outdoor sports, if they can effectively regulate their emotional responses to fatigue, frustration, or discomfort, they are more likely to maintain enthusiasm and long-term engagement in the activity (Wicks et al., 2022). Especially in collective or more challenging sports, self-regulation ability helps participants improve their performance and cohesion, as well as achieve a sense of accomplishment and psychological satisfaction (Collins and Durand-Bush, 2010). For university students, outdoor sports not only serve as a form of physical activity but also as an important means of regulating emotions and buffering external pressures (Wang T. et al., 2025). In this process, self-regulation ability may play a role in the relationship between outdoor sports and learning burnout. By regulating emotions, stabilizing motivation, and strengthening goal awareness, self-regulation ability helps students recover psychological resources after outdoor sports, enabling them to more effectively deal with the stress reactions generated during their learning process.

*H3*: Self-regulation statistically mediates the association between outdoor sports and learning burnout.

### 2.3 Chain mediation effect

Learning burnout is a negative psychological state resulting from long-term academic load, characterized by emotional exhaustion, lack of motivation, and a decline in learning effectiveness (Hongyi et al., 2023; Jianwen et al., 2022). Research indicates that sustained academic pressure weakens an individual’s emotional regulation ability, disrupts cognitive functions, and reduces their engagement with learning tasks (Teixeira et al., 2021). If students lack effective internal regulation mechanisms, they are often unable to recover from stress and are more likely to fall into burnout. Individuals with good self-regulation abilities can proactively identify emotional changes and adopt appropriate strategies to adjust their state, thereby maintaining goal-oriented behavior and relatively stable psychological states.

When facing continuous academic challenges, students typically rely on social support systems to alleviate stress and maintain psychological balance. Studies have found that understanding, encouragement, and guidance from family members, peers, and teachers can significantly reduce feelings of loneliness and anxiety (Abrams et al., 2022; Pineda et al., 2022). Social support helps individuals establish cognitive frameworks for stressors by conveying positive emotions and practical information, while also enhancing their confidence and problem-solving abilities. Through receiving support, students gradually improve their control over emotions and behaviors, thus forming more adaptive self-regulation pathways.

Meanwhile, physical activity has a positive impact on individuals’ psychological regulation abilities, particularly outdoor sports that take place in natural environments. Research shows that regular physical activity promotes the recovery of the nervous system, activates emotional regulation mechanisms, and enhances individuals’ ability to adapt to environmental changes (Smits et al., 2008; Yan et al., 2020). While participating in outdoor sports, students not only release accumulated emotional stress but also build new social connections through interaction. This process enhances their sense of social belonging and further strengthens their emotional regulation and behavioral control abilities, providing psychological resources to more effectively cope with academic tasks.

*H4*: Social support and self-regulation sequentially mediate the association between outdoor sports and learning burnout.

### 2.4 Theoretical model

**Figure 1:**
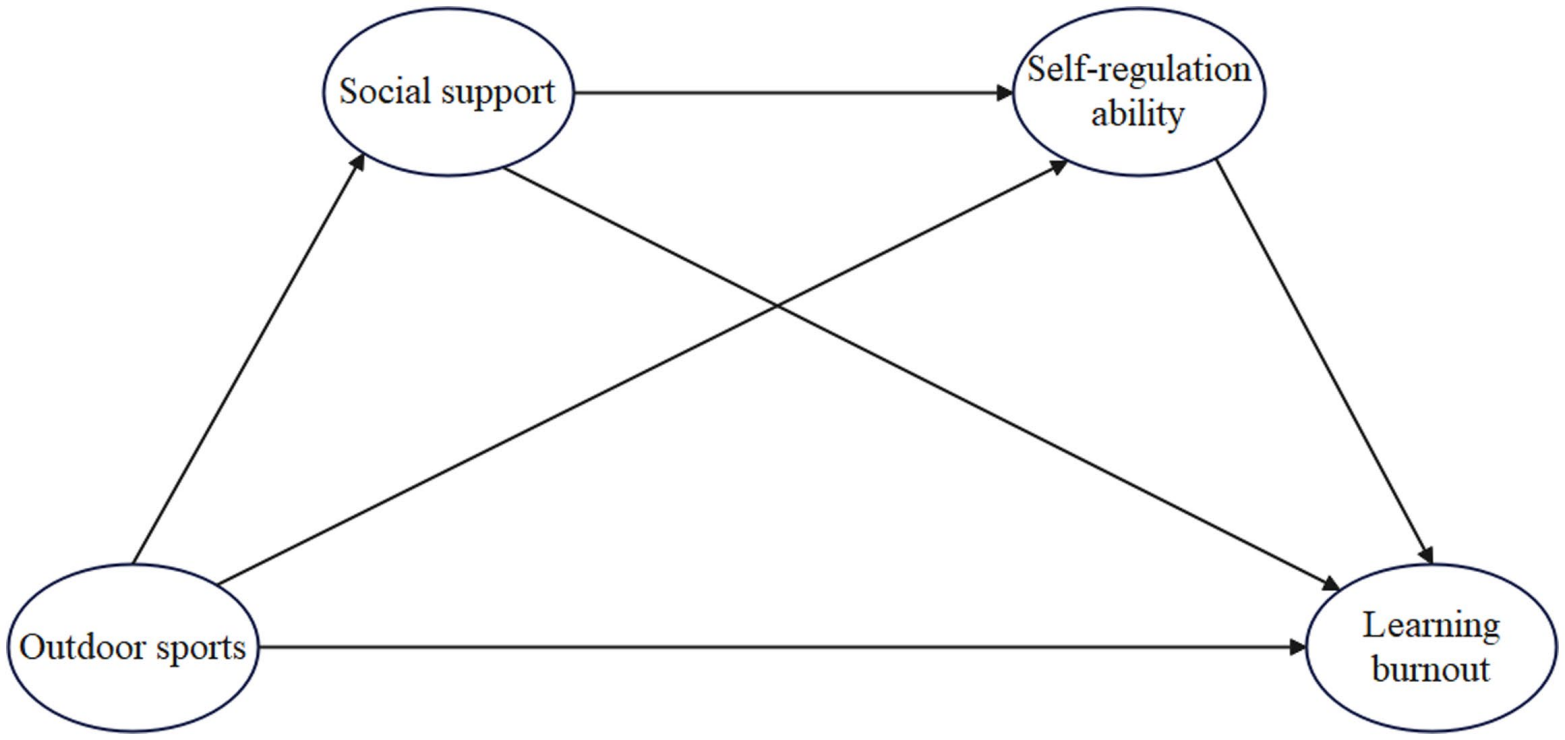
Theoretical model of the associations between outdoor sports and college students’ learning burnout: the multiple mediation of social support and self-regulation.

This study constructs a relational framework (see Figure 1) to examine how outdoor sports are associated with college students’ learning burnout through social support and self-regulation. The research focuses on: (Abrams et al., 2022) the direct association between outdoor sports and learning burnout; (Acoba, 2024) the statistical mediation of social support and self-regulation in these associations; (Ahn et al., 2016) and the sequential mediation pathway whereby outdoor sports relate to greater perceived social support and, in turn, to stronger self-regulation, together linked to lower reported burnout.

The model hypothesizes that outdoor sports not only directly influence college students’ levels of learning burnout but also have an indirect effect through the two variables of social support and self-regulation ability. In this pathway, social support may enhance self-regulation ability, guiding students to form more positive coping strategies, thereby alleviating learning burnout. By constructing this theoretical framework, the study provides a multi-level perspective for understanding the mechanisms through which outdoor sports affect learning burnout and offers theoretical support and path references for universities to implement student psychological interventions and health promotion practices.

## 3 Materials and methods

### 3.1 Sample estimation and data collection

To ensure the appropriateness of the sample, this study followed a commonly used rule of thumb in social science research, namely that the sample size should be 5–10 times the total number of questionnaire items (Jeffrey and Hoogland, 1998). A stratified sampling method was adopted, and college students were recruited from multiple universities across Sichuan Province, Chongqing Municipality, and Guizhou Province in China, covering all four academic years from freshman to senior. This design not only guaranteed regional diversity but also included students at different stages of study, thereby enhancing the breadth and representativeness of the sample. In total, 1,000 students were selected as participants.

The survey was conducted using paper-based questionnaires, administered in classroom settings under the guidance of trained teachers, with completion time controlled within 15 min. All participants took part voluntarily with informed consent. The questionnaire included four instruments: the physical exercise scale, the learning burnout scale, the social support scale, and the self-regulation ability scale. A total of 1,000 questionnaires were distributed, 976 were collected, yielding a response rate of 97.60%.

To ensure data quality and methodological rigor, we established explicit rules for data screening. Specifically: (1) questionnaires with more than 20.00% missing responses on key items were excluded; (2) responses with logical errors or clearly unreasonable patterns (e.g., all extreme values within one scale) were removed; and (3) questionnaires showing obvious patterned responses (e.g., the same option selected for all items) were discarded. Following this process, 34 invalid questionnaires were excluded, resulting in 942 valid responses and an effective response rate of 96.52%. The final sample was relatively balanced in terms of gender, academic year distribution, and regional coverage, thus providing a reasonably representative basis for subsequent statistical analyses.

### 3.2 Measurement

All measurement tools used in this study were based on existing validated scales with good reliability and validity. Below are the specific measurement tools and their related information.

#### 3.2.1 Outdoor sports

The physical exercise scale, developed by Liang (1994), consists of three dimensions—exercise intensity, duration, and frequency—with a total of three items, rated on a five-point Likert scale. In the present study, the scale demonstrated good internal consistency (Cronbach’s *α* = 0.777). Confirmatory factor analysis indicated a good model fit (*χ*^2^/df = 4.611, CFI = 0.980, TLI = 0.970, RMSEA = 0.062, 90% CI [0.051, 0.074]).

#### 3.2.2 Learning burnout

The Learning Burnout Scale, developed by Lian et al. (2005), comprises three dimensions—emotional exhaustion, behavioral withdrawal, and reduced sense of accomplishment—with a total of 20 items, rated on a five-point Likert scale. In the present study, the scale demonstrated high reliability (Cronbach’s *α* = 0.939). Confirmatory factor analysis indicated a good model fit (*χ*^2^/df = 2.656, CFI = 0.976, TLI = 0.973, RMSEA = 0.042, 90% CI [0.037, 0.047]).

#### 3.2.3 Social support

The social support scale, revised by Chen et al. (2016), consists of three dimensions—family support, friend support, and other support—with a total of 12 items, rated on a five-point Likert scale. In the present study, the scale demonstrated good reliability (Cronbach’s *α* = 0.913). Confirmatory factor analysis indicated good structural validity (*χ*^2^/df = 4.757, CFI = 0.972, TLI = 0.963, RMSEA = 0.063, 90% CI [0.055, 0.071]).

#### 3.2.4 Self-regulation ability

The self-regulation ability scale, developed by Kong and Haidong (2012), comprises seven dimensions—self-efficacy, metacognitive strategies, cognitive strategies, external motivation, emotional regulation, intrinsic motivation, and cooperative learning—with a total of 31 items, rated on a five-point Likert scale. In the present study, the scale demonstrated high reliability (Cronbach’s *α* = 0.942). Confirmatory factor analysis indicated a good model fit (*χ*^2^/df = 1.775, CFI = 0.978, TLI = 0.977, RMSEA = 0.029, 90% CI [0.025, 0.032]).

Table 1 summarizes the measurement scales used in this study, along with specific information such as the number of items and scoring range.

**Table 1:** Scales used in this study.

Scale	Author (year)	Item quantity	Scoring	Dimensions
Physical exercise	Liang (1994)	3	5	Exercise intensity; duration; exercise frequency
Learning burnout	Lian et al. (2005)	20	5	Emotional exhaustion; behavioral withdrawal; reduced sense of accomplishment
Social support	Chen et al. (2016)	12	5	Family; friends; others
Self-regulation ability	Kong and Haidong (2012)	31	5	Self-efficacy; metacognitive strategies; cognitive strategies; external motivation; emotional regulation; intrinsic motivation; cooperative learning

### 3.3 Data analysis procedure

This study used SPSS 26.0 and AMOS 24.0 software for data analysis. First, the Harman single-factor test was employed to assess common method bias. The results showed that the variance explained by a single factor was less than 40%, indicating that there was no serious common method bias in the data, making it suitable for subsequent analysis (Tang and Wen, 2020). Next, the means and standard deviations of outdoor sports, learning burnout, social support, and self-regulation ability were calculated, and Pearson correlation analysis was conducted to examine the associations among the variables (Zhu et al., 2014).

To further explore gender and grade differences in the main variables, independent-samples *t* tests and one-way analysis of variance (ANOVA) were conducted for statistical comparison (Fang and Zhang, 2012). Subsequently, structural equation modeling (SEM) was employed to test the hypothesized multiple mediation model, focusing on the mediating roles of social support and self-regulation ability in the relationship between outdoor sports and learning burnout in college students. Model fit was evaluated using standard indices, including *χ*^2^/df, CFI, TLI, SRMR, and RMSEA. In accordance with established SEM practice, RMSEA was reported with a 90% confidence interval because close-fit hypotheses are conventionally evaluated with one-sided tests at *α* = 0.05, and the corresponding interval is defined at the 90% level. Path analysis was then applied to estimate direct and indirect effects, and the significance of the indirect effects was tested with 2,000 bootstrap resamples using bias-corrected 95% confidence intervals (Wen et al., 2004; Wen and Ye, 2014).

This analytic strategy ensured that both model fit evaluation and effect estimation followed recognized methodological standards, thereby enhancing the robustness and validity of the results.

## 4 Results

### 4.1 Common method bias test

To assess the potential impact of common method bias, Harman’s single-factor test was conducted. All measurement items were subjected to an exploratory factor analysis without rotation. The results showed that a total of 14 factors had eigenvalues greater than 1, and the first factor accounted for 30.64% of the total variance. Since this percentage is below the critical threshold of 40.00%, it suggests that common method bias is not a serious concern in this study and does not significantly distort the observed relationships among variables.

### 4.2 Descriptive statistics, reliability, and model fit

Table 2 presents the means, standard deviations, internal consistency coefficients (Cronbach’s *α*), and the fit indices of the confirmatory factor analyses (CFA) for each key variable. All constructs exhibited acceptable to excellent levels of internal consistency reliability, with Cronbach’s *α* values ranging from 0.777 to 0.942. The CFA results demonstrated that all measurement models achieved acceptable levels of model fit. Specifically, the *χ*^2^/df values for all variables were below the threshold of 5.0, indicating reasonable model fit. The comparative fit index (CFI) and Tucker–Lewis index (TLI) for all variables exceeded the conventional cutoff of 0.950, suggesting good model fit. Additionally, the standardized root mean square residual (SRMR) values were all below 0.060, and the root mean square error of approximation (RMSEA) values were within the acceptable range (i.e., below 0.080), with 90% confidence intervals falling within narrow and theoretically justifiable bounds. These results provide robust evidence for the convergent validity and reliability of the measurement instruments.

**Table 2:** Descriptive statistics, internal consistency reliability, and fit indices for confirmatory factor analysis (CFA) of key variables.

Variable	*M*	*SD*	*α*	*χ*^2^/df	CFI	TLI	SRMR	RMSEA (90% CI)
Outdoor sports	3.624	0.802	0.777	4.611	0.980	0.970	0.024	0.062 (0.051–0.074)
Social support	3.702	0.775	0.913	4.757	0.972	0.963	0.027	0.063 (0.055–0.071)
Self-regulation ability	3.587	0.693	0.942	1.775	0.978	0.977	0.028	0.029 (0.025–0.032)
Learning burnout	3.375	0.828	0.939	2.656	0.976	0.973	0.024	0.042 (0.037–0.047)

### 4.3 Correlation analysis

Pearson correlation analysis was conducted to examine the relationships among the key variables in the proposed model, including outdoor sports, social support, self-regulation ability, and learning burnout. The results are presented in Table 3. As shown, outdoor sports were positively correlated with social support (*r* = 0.499, *p* < 0.001) and self-regulation ability (*r* = 0.473, *p* < 0.001), and negatively correlated with learning burnout (*r* = −0.452, *p* < 0.001). Social support was positively associated with self-regulation ability (*r* = 0.594, *p* < 0.001), and negatively associated with learning burnout (*r* = −0.512, *p* < 0.001). Likewise, self-regulation ability was significantly negatively correlated with learning burnout (*r* = −0.590, *p* < 0.001). These results suggest that higher levels of outdoor sports participation, social support, and self-regulation ability are all significantly associated with lower levels of learning burnout. Furthermore, the significant positive associations among outdoor sports, social support, and self-regulation ability support the hypothesized mediation pathways for further testing via structural equation modeling.

**Table 3:** Correlation coefficient of key variables.

Variable	Outdoor sports	Social support	Self-regulation ability	Learning burnout
Outdoor sports	1			
Social support	0.499^***^	1		
Self-regulation ability	0.473^***^	0.594^***^	1	
Learning burnout	−0.452^***^	−0.512^***^	−0.590^***^	1

^***^p < 0.001, two-tailed.

### 4.4 Differences across gender and grade levels

Independent-samples *t*-tests and one-way ANOVAs were conducted to examine demographic differences in the four key variables (outdoor sports, social support, self-regulation ability, and learning burnout). The results are presented in Table 4.

**Table 4:** Descriptive statistics and differences across demographic variables for key variables.

Demographic variables	Category (*n*)	Outdoor sports		Social support		Self-regulation ability		Learning burnout	
		*M*	*SD*	*M*	*SD*	*M*	*SD*	*M*	*SD*
Gender	Male (472)	3.793	0.757	3.857	0.728	3.702	0.664	3.431	0.817
	Female (470)	3.455	0.810	3.547	0.790	3.473	0.702	3.319	0.836
	*t*	6.611		6.275		5.145		2.072	
	*p*	<0.001		< 0.001		< 0.001		0.039	
	Cohen’s *d*	0.431		0.409		0.335		0.315	
Grade	Freshman (242)	3.963	0.710	3.463	0.751	3.358	0.663	3.172	0.810
	Sophomore (231)	3.654	0.762	3.612	0.798	3.500	0.718	3.356	0.839
	Junior (238)	3.549	0.829	3.792	0.791	3.647	0.708	3.413	0.866
	Senior (231)	3.318	0.769	3.951	0.667	3.855	0.575	3.569	0.747
	*F*	28.782		18.798		23.762		9.504	
	*p*	<0.001		<0.001		<0.001		<0.001	
	Partial *η^2^*	0.084		0.057		0.071		0.029	

For gender, male students reported significantly higher levels of outdoor sports participation (*t* = 6.611, *p* < 0.001, Cohen’s *d* = 0.431), social support (*t* = 6.275, *p* < 0.001, Cohen’s d = 0.409), and self-regulation ability (*t* = 5.145, *p* < 0.001, Cohen’s *d* = 0.335) compared with female students. In contrast, learning burnout was significantly higher among males (*t* = 2.072, *p* = 0.039, Cohen’s *d* = 0.135).

For grade, significant differences were found across the four cohorts. Outdoor sports participation declined steadily from freshmen (*M* = 3.963, SD = 0.710) to seniors (*M* = 3.318, SD = 0.769), *F* = 28.782, *p* < 0.001, partial *η^2^* = 0.084. Social support increased progressively with grade level, with seniors reporting the highest scores (*M* = 3.951, SD = 0.667), *F* = 18.798, *p* < 0.001, partial *η^2^* = 0.057. Similarly, self-regulation ability showed an upward trend, with seniors scoring the highest (*M* = 3.855, SD = 0.575), *F* = 23.762, *p* < 0.001, partial *η^2^* = 0.071. Learning burnout also varied significantly by grade, with seniors reporting the greatest level (*M* = 3.569, SD = 0.747), *F* = 9.504, *p* < 0.001, partial *η^2^* = 0.029. *Post hoc* comparisons (Bonferroni) confirmed that differences were significant across most adjacent grade groups.

### 4.5 Model fit and mediation analysis

**Figure 2:**
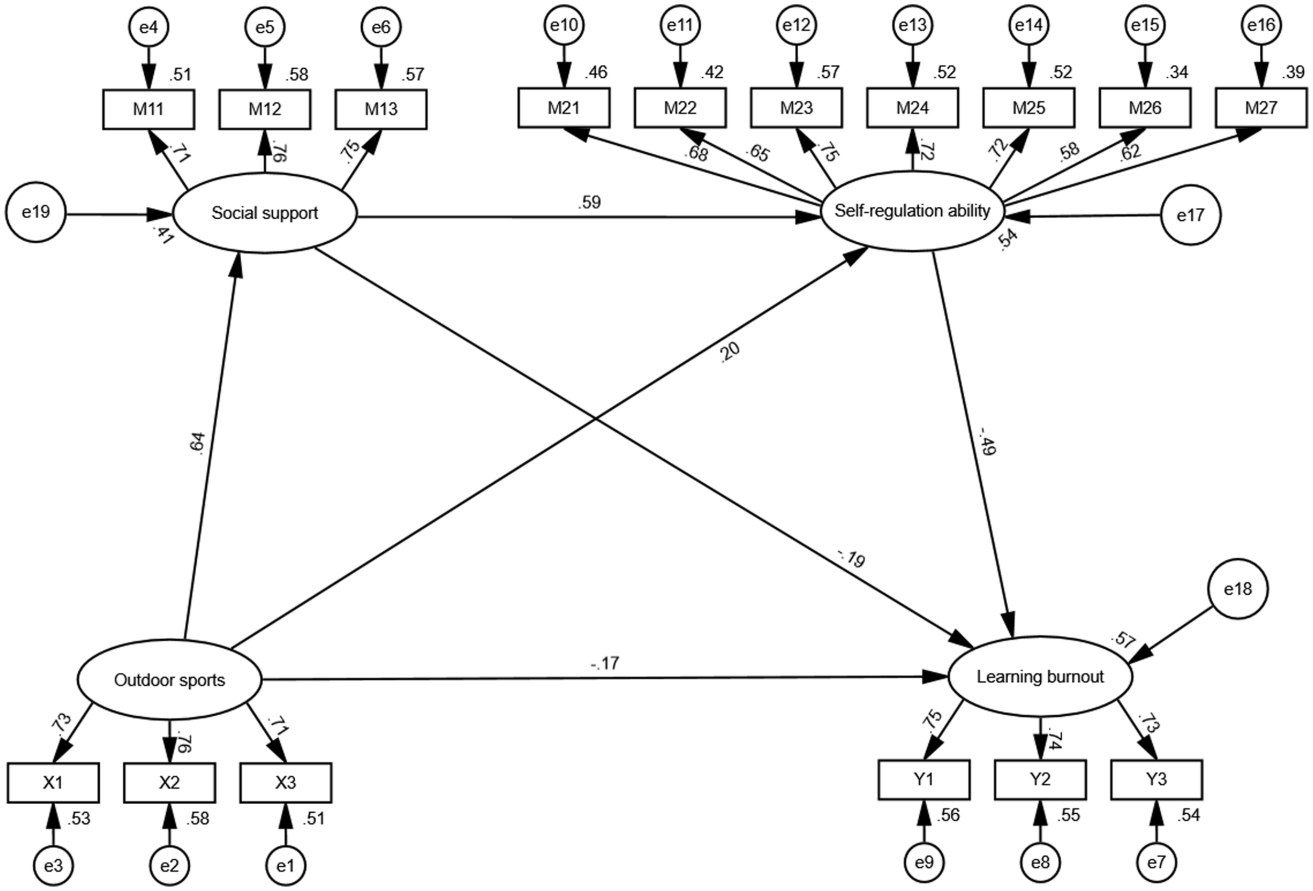
Structural equation model. All paths are significant at the 0.01 level.

Structural equation modeling (SEM) was employed to test the hypothesized relationships among outdoor sports, social support, self-regulation ability, and learning burnout. The model demonstrated a good overall fit to the data: *χ*^2^/df = 2.702, CFI = 0.972, TLI = 0.966, SRMR = 0.032, and RMSEA = 0.043 (90% CI: 0.036–0.049), all of which fall within the acceptable thresholds, indicating that the hypothesized model fits the observed data well (see Table 5 and Figure 2).

**Table 5:** Questionnaire model fitting indicators.

Model fit	*χ*^2^/df	CFI	TLI	SRMR	RMSEA (90%CI)
Model	2.702	0.972	0.966	0.032	0.043 (0.036–0.049)

All path coefficients in the structural model were significant at the 0.01 level (see Table 6). Specifically, outdoor sports showed a significant direct negative association with learning burnout (*β* = −0.170, *p* = 0.001), supporting Hypothesis H1. This result indicates that increased engagement in outdoor sports is associated with a decrease in reported levels of learning burnout among college students.

**Table 6:** Total, direct and indirect effects in the multiple mediator model.

Path	*β*	Boot SE	*p*	Boot LLCI	Boot ULCI	Ratio
Direct effect						
OS → LB	−0.170	0.051	0.001	−0.267	−0.070	29.72%
All indirect effects						70.29%
OS → SS → LB	−0.121	0.042	0.005	−0.209	−0.042	21.16%
OS → SA → LB	−0.097	0.026	0.001	−0.147	−0.051	16.96%
OS → SS → SA → LB	−0.184	0.027	0.001	−0.242	−0.139	32.17%
Total effect	−0.572	0.032	< 0.001	−0.633	−0.509	100%

OS, outdoor sports; SS, social support; SA, self-regulation ability; LB, learning burnout. Boot LLCI, the lower bound of the 95% confidence interval. Boot ULCI, the upper limit of the 95% confidence interval (percentile bootstrap method with bias correction). The bootstrap sample size is set at 2,000.

The total indirect effect of outdoor sports on learning burnout was significant, accounting for 70.29% of the total effect. Three mediation pathways were identified. First, social support partially mediated the association between outdoor sports and learning burnout (*β* = −0.121, 95% CI [−0.209, −0.042], *p* = 0.005), supporting Hypothesis H2; higher levels of outdoor sports participation were associated with greater perceived social support, which related to lower reported burnout. Second, self-regulation also served as a partial mediator (*β* = −0.097, 95% CI [−0.147, −0.051], *p* = 0.001), supporting Hypothesis H3; greater engagement in outdoor sports was associated with stronger self-regulatory capacities, which related to lower burnout. Third, a significant sequential mediation pathway was observed: outdoor sports were associated with higher social support, which related to stronger self-regulation, together linked to lower reported burnout (*β* = −0.184, 95% CI [−0.242, −0.139], *p* < 0.001). This sequential pathway accounted for the largest proportion of the total indirect effect (32.17%), providing statistical support for Hypothesis H4.

The total effect of outdoor sports on learning burnout was −0.572 (95% CI [−0.633, −0.509], *p* < 0.001), indicating a substantial overall relationship. Among the three mediating paths, the sequential mediation involving both social support and self-regulation ability contributed the most to this total effect, highlighting the joint importance of interpersonal and intrapersonal resources in the mechanism through which outdoor sports alleviate academic exhaustion.

## 5 Discussion

### 5.1 Demographic differences

Gender- and grade-based heterogeneity in social support, psychological resources, and exercise behaviors emerged clearly and is broadly consonant with extant evidence while clarifying several China-specific pathways (Sheng et al., 2025). Reports of higher perceived support, resilience, self-efficacy, and exercise adherence among male students are compatible with work showing that sport contexts scaffold peer interaction, confidence, and goal orientation—mechanisms that may advantage males when participation is more frequent or more challenge-oriented (Li et al., 2025). Reviews and meta-analytic syntheses further underscore the centrality of social support for adjustment and well-being in youth and university settings, reinforcing the plausibility of gender-linked differences where support networks are differentially mobilized through activity participation. In Chinese samples, teacher and peer support relate robustly to mental well-being, and perceived support predicts physical activity via exercise self-efficacy, suggesting a cascading route from support to behavior to resource accrual that aligns with the present pattern (Zhou et al., 2024; Su and Liu, 2025). Evidence from collegiate athletes also links higher perceived support to lower burnout-related risk, which offers a parallel mechanism relevant to outdoor-activity contexts on campus.

Across academic standing, the combination of higher perceived support among first-year students and stronger internal resources among seniors maps onto a developmental trajectory in which external scaffolds are salient during transition but give way to consolidated regulatory capacities with accumulated academic cycles and performance contingencies (Xiong et al., 2025). Adventure- and nature-based programs in emerging adults have been shown to strengthen well-being and connection with nature, providing a credible experiential substrate for the upward trends in resilience and self-efficacy observed at later grades (Puhakka, 2023). Complementary work in Chinese secondary and tertiary contexts indicates that social support operates as a mediator between participation and academic stress or burnout, again consistent with the notion that sustained engagement progressively internalizes coping skills and stabilizes exercise habits as students advance through university (Chen et al., 2023; Li et al., 2025; Li and Hao et al., 2025). Collectively, the demographic gradients documented here cohere with theory and evidence on socially embedded activity and resource development, and they motivate tailored provision of outdoor opportunities that address gender-specific barriers and stage-specific needs in higher education settings.

### 5.2 Direct relationships

The structural equation modeling confirmed a significant direct effect of outdoor sports on learning burnout (*β* = −0.17, *p* = 0.001), supporting H1. This finding is consistent with a broad body of research showing that physical activity in outdoor or natural environments is associated with reductions in stress, anxiety, and exhaustion, functioning as a potential protective factor for students’ academic well-being (Bramwell et al., 2023). At the same time, the size of the direct effect observed here is more modest than that reported in some experimental or intervention studies, where structured outdoor programs often yield stronger immediate associations with psychological outcomes (Stefanica et al., 2025; Wang W. et al., 2025). The discrepancy may reflect differences in research design, cultural context, and the heterogeneity of exercise behaviors in real-world student populations.

Several mechanisms may help explain the observed direct effect. Outdoor activity provides opportunities for attentional restoration, enabling recovery of depleted cognitive resources; it may also facilitate emotion regulation by lowering physiological arousal and supporting adaptive coping in high-stress contexts (Wicks et al., 2022). In addition, participation in outdoor sports creates experiential settings that reinforce a sense of control, autonomy, and competence, thereby contributing to stronger self-perceived learning efficacy and lower emotional exhaustion. Evidence from Chinese university samples further corroborates these patterns, showing that outdoor and group-based activities are associated with improvements in engagement and declines in burnout-related symptoms (Sun et al., 2023).

Although statistically robust, the direct path accounted for only 29.72% of the total effect, indicating that the association between outdoor sports and learning burnout was transmitted predominantly through indirect channels. This finding aligns with prior reports that the benefits of physical activity are frequently explained by psychosocial mechanisms, such as perceived social support and self-regulation (Eime et al., 2013). The following section therefore turns to a detailed examination of these mediation pathways.

### 5.3 Mediation effects

The multiple mediation analysis revealed that social support and self-regulation ability served as significant mediators in the association between outdoor sports and learning burnout, lending empirical support to H2, H3, and H4. A total of 70.29% of the effect of outdoor sports on burnout was transmitted through indirect pathways, underscoring the centrality of psychological and social mechanisms in explaining why outdoor physical activity alleviates academic exhaustion.

The first indirect pathway was through social support (*β* = −0.121, 95% CI [−0.209, −0.042], *p* = 0.005), consistent with evidence that physical activity embedded in group or peer contexts enhances perceived emotional and instrumental support (Spruijtenburg et al., 2025). Prior studies have shown that students who regularly participate in organized outdoor activities report stronger interpersonal bonds and higher levels of mutual encouragement, both of which reduce vulnerability to academic strain (Bramwell et al., 2023). This result aligns with the buffering hypothesis of support, which posits that supportive networks moderate the impact of stressors and help preserve psychological health in high-demand environments.

The second indirect pathway was through self-regulation ability (*β* = −0.097, 95% CI [−0.147, −0.051], *p* = 0.001), confirming H3. This pattern is compatible with prior findings that outdoor sports fosters self-regulatory capacities such as goal-setting, persistence, and adaptive coping (Neill and Dias, 2001; Sibthorp and Jostad, 2014). Empirical studies in student samples have indicated that emotionally stimulating yet manageable physical challenges cultivate regulatory skills, enabling more effective responses to fatigue, frustration, and workload pressures. The present result adds further support to the view that self-regulation operates as a pivotal psychological resource through which physical activity translates into academic resilience.

The strongest effect was observed in the sequential pathway: outdoor sports → social support → self-regulation → learning burnout (*β* = −0.184, 95% CI [−0.242, −0.139], *p* < 0.001), validating H4. This path accounted for 32.17% of the total effect and reflects a layered mechanism wherein interpersonal resources facilitate the development of intrapersonal regulatory capacities, which together buffer against burnout. This pattern corresponds with the resource caravan principle in the conservation of resources framework, which holds that psychosocial resources often accumulate and operate synergistically to protect individuals under conditions of chronic stress (Hobfoll, 1989). Similar resource-accumulation processes have been documented in university and sport contexts, where supportive networks amplify efficacy and regulation, thereby producing downstream benefits for performance and well-being.

Taken together, the mediation results indicate that social support and self-regulation statistically account for a substantial share of the association between outdoor sports and learning burnout. The sequential pathway suggests that interpersonal resources and intrapersonal regulation operate in tandem, describing how these variables are linked in the present data without implying causal ordering.

### 5.4 Theoretical and practical implications

#### 5.4.1 Theoretical implications

This study advances theoretical understanding of the relationship between physical activity and academic burnout by clarifying the distinctive role of outdoor sports. Prior research has generally affirmed that exercise benefits mental health, yet much of it has treated activity as a uniform category. The present findings highlight that outdoor activities exert a particularly strong protective effect because they combine physical exertion with exposure to natural environments and opportunities for social interaction. This dual context distinguishes outdoor sports from non-outdoor forms of exercise and explains why their effects extend beyond the well-recognized contributions of general physical activity.

A second theoretical contribution lies in identifying the multiple mediation pathways through which outdoor sports influence burnout. Specifically, outdoor activities enhance perceived social support and foster self-regulation, and these two mechanisms operate both independently and sequentially. This integrated framework demonstrates that outdoor sports serve as catalysts for mobilizing interpersonal and intrapersonal resources simultaneously. In doing so, the study moves beyond the general assertion that exercise is beneficial, offering a more precise explanation of how and why outdoor sports are uniquely effective in reducing student burnout.

#### 5.4.2 Practical implications

The results also carry practical significance for higher education institutions. The unique features of outdoor sports—natural environmental exposure and embedded social contexts—make them particularly effective in alleviating burnout. Universities should therefore recognize outdoor sports not merely as recreational activity but as a strategic component of student development and mental health support.

First, program design should deliberately balance group-based and individual outdoor activities. Group-based formats, such as team hiking, outdoor cooperative challenges, and campus sport festivals, are especially effective in strengthening peer support networks and reducing feelings of isolation. In contrast, individual outdoor activities, such as running, cycling, or walking in natural settings, may be more closely tied to strengthening autonomy, focus, and self-regulation. Recognizing the complementary benefits of these formats allows institutions to maximize the impact of outdoor programs.

Second, the demographic patterns observed in this study point to the value of tailored interventions. Female students may require confidence-building and inclusive outdoor opportunities to address lower perceived support and self-efficacy. First-year students may benefit from outdoor activities that ease transitional stress and facilitate early social integration, while senior students can be encouraged to assume leadership roles in organizing or guiding outdoor programs, thereby consolidating their self-regulatory skills and autonomy.

Third, the findings have direct implications for educational administrators. Universities can incorporate structured outdoor activities into student development curricula and link them with counseling and academic support services. Policies that expand access to safe and engaging outdoor spaces, strengthen interdepartmental collaboration between physical education units and student affairs, and provide institutional support for outdoor sports programs can ensure that these activities become sustainable tools for reducing burnout and promoting holistic well-being.

## 6 Limitations and future directions

### 6.1 Limitations

Despite the theoretical contributions and empirical robustness of this study, several limitations should be acknowledged, which in turn point to important directions for future research. First, the study employed a cross-sectional research design, which limits the ability to draw causal inferences regarding the relationships among outdoor sports, social support, self-regulation ability, and learning burnout. Although the proposed model was theoretically grounded and statistically supported through structural equation modeling and bootstrapped mediation analysis, the directionality of effects cannot be definitively established. It is possible, for instance, that students with lower levels of learning burnout are more motivated to engage in outdoor activities, or that higher self-regulation promotes greater participation in physical exercise. Future research should therefore employ longitudinal or experimental designs to clarify temporal ordering and provide stronger evidence for causal mechanisms.

Second, the data were collected through self-report questionnaires, which may introduce common method variance, despite the results of Harman’s single-factor test suggesting that such bias is not substantial. Self-report measures are susceptible to social desirability effects and recall bias, particularly when assessing constructs like exercise adherence, emotional regulation, and perceived burnout. The inclusion of multi-informant assessments, behavioral indicators, or physiological data (e.g., activity tracking, cortisol levels) in future studies would enhance measurement accuracy and validity.

Third, while the study sample was geographically diverse—spanning three provinces—it consisted solely of Chinese university students, which may limit the generalizability of the findings to other cultural or educational contexts. Cultural values influence perceptions of social support, coping styles, and help-seeking behavior. For example, collectivist norms may shape the way students interpret and engage with social support networks in outdoor settings. Future research could extend the current model to international or cross-cultural samples to examine the cultural invariance of the proposed mechanisms and explore whether similar resource pathways operate in more individualistic or alternative academic environments.

Fourth, the study focused on outdoor sports as a general construct, without differentiating between types, intensity, frequency, or context (e.g., individual vs. team, competitive vs. recreational, natural vs. built environment). Given that different modalities of physical activity may exert distinct psychological and social effects, future studies should adopt a more nuanced categorization of outdoor activities to examine which characteristics are most effective in reducing learning burnout and enhancing psychological resources.

Finally, while the current model incorporated two critical mediators—social support and self-regulation ability—it is likely that other psychological constructs also play important roles in this process. Variables such as self-esteem, mindfulness, academic motivation, or perceived autonomy may further mediate or moderate the relationship between outdoor sports and learning outcomes. Expanding the model to include additional theoretically relevant constructs would enrich our understanding of the full spectrum of mechanisms at play.

## 7 Conclusion

This study found that outdoor sports were associated with lower reported levels of learning burnout among university students, with social support and self-regulation statistically accounting for a substantial proportion of this association, including a sequential pathway linking the two mediators. These results contribute to a more nuanced understanding of how physical, social, and psychological resources are interrelated in academic contexts and may provide reference points for the design of campus-based programs to support student well-being. Given the cross-sectional nature of the data, the observed patterns should be interpreted as correlational rather than causal, and further longitudinal or experimental studies are needed to validate the temporal direction and underlying mechanisms of these associations.

## Data Availability

The original contributions presented in the study are included in the article/supplementary material, further inquiries can be directed to the corresponding author.
